# Effects of two triterpenoids from *Nigella sativa* seeds on insulin resistance of 3T3-L1 adipocytes

**DOI:** 10.3389/fnut.2022.995550

**Published:** 2022-08-23

**Authors:** Jinfeng Wei, Baoguang Wang, Yixiao Chen, Qiuyi Wang, Adel F. Ahmed, Lili Cui, Xuefeng Xi, Wenyi Kang

**Affiliations:** ^1^National R&D Center for Edible Fungus Processing Technology, Henan University, Kaifeng, China; ^2^Shenzhen Research Institute of Henan University, Shenzhen, China; ^3^Medicinal and Aromatic Plants Researches Department, Horticulture Research Institute, Agricultural Research Center, Giza, Egypt; ^4^College of Physical Education, Henan University, Kaifeng, China; ^5^Joint International Research Laboratory of Food & Medicine Resource Function, Kaifeng, China

**Keywords:** *Nigella sativa* seeds, triterpenoids, 3T3-L1 cells, insulin resistance, IRS/AKT/PI-3K

## Abstract

Insulin resistance (IR) is a physiological abnormality that occurs when insulin fails to activate the signal transduction pathway in target organs. It was found that supplementation of *Nigella sativa* seeds with oral antidiabetic medicines helps improve blood glucose control by enhanced β cells activity and alleviation of IR. However, the activities and related mechanisms of phytochemicals from *N. sativa* seeds have not been thoroughly explored. In this study, the effects of two triterpenoids, 3-*O*-[β-D-xylopyranose-(1→3)-α-L-rhamnose-(1→2)-α-L-arabinose]-28-*O*-[α-L-rhamnose-(1→4)-β-D-glucopyranose-L-(1→6)-β-D-glucopyranose]-hederagenin (Hxrarg) and 3-*O*-[β-D-xylopyranose-(1→3)-α-L-rhamnose-(1→2)-α-L-arabinose]-hederagenin (Hxra), on IR were studied by 3T3-L1 adipocytes model. The results demonstrated that Hxrarg and Hxra inhibited maturation of 3T3-L1 preadipocytes, dramatically stimulated glucose uptake of IR-3T3-L1 adipocytes, promoted transcription of IRS, AKT, PI-3K, and GLUT4 mRNA. Western Blot results suggested that Hxrarg and Hxra were able to markedly up-regulate expression of p-IRS, p-AKT, PI-3K, and GLUT4 proteins. These findings could provide a basic foundation for the continued development and application of *N. sativa* in medicine and functional foods.

## Introduction

The concept of “nutritious food” or “functional food” has progressively gained popularity in recent decades (1, 2). People have conducted substantial and in-depth study on healthy and useful traditional foods due to the growth of the global functional food market. Many modest but essential substances with extensive biological functions can be found among many functional food materials, including herbs and spices (3).

*Nigella sativa* is famous for its culinary applications with high nutritive value and medicinal value (4). In traditional medicine, essential oil, powder, or extract of *N. sativa* seeds have antioxidant, immunomodulatory, anti-inflammatory, and antimicrobial effects (5). Many ailments are treated with them, including asthma, bronchitis, rheumatism, headaches, and hypertension. Surprisingly, several researches have demonstrated that *N. sativa* seeds have the ability to treat SARS-CoV-2, the infection responsible for the recent COVID-19 pandemic (6, 7).

However, the therapeutic potential of *N. sativa* seeds for different diseases has not been explored equally. Diabetes mellitus (DM) is a metabolic illness. Type 2 diabetes mellitus (T2DM) accounts for 90% of all diabetes cases. Insulin resistance (IR) is assumed to be the physiological and pathological basis of T2DM, occurring 10 to 15 years before T2DM. The onset and progression of IR causes a decrease in the sensitivity and reactivity of target tissues to insulin, as well as an increase in endogenous insulin to compensate (8). Weight growth is associated with increased endogenous insulin levels, which exacerbates IR (9, 10). This vicious cycle continues until pancreatic cell activity is unable to adequately fulfill the insulin demand generated by IR, resulting in hyperglycemia.

Adipose tissue is a critical insulin target and the principal site of glucose and lipid metabolism, it is also a factor in the development of IR and T2DM. There’s evidence that aberrant adipose tissue function can raise circulating triglyceride (TG) and fatty acid levels, causing IR (11). Furthermore, excessive lipid secretion in adipose tissue reduces peripheral tissue insulin sensitivity and interferes with insulin signal pathway defects at several levels, including intracellular insulin receptor, phosphorylation of insulin receptor substrate (IRS) and post-receptor signal transduction, resulting in IR, blood glucose elevation and diabetes (12).

Currently, *N. sativa* is being exploited as a diabetic therapy drug. *N. sativa* seeds and preparations are commonly used as auxiliary medications to treat diabetes and its consequences. Bamosa discovered that taking 2 g of *N. sativa* seeds daily with anti-DM medications reduced IR considerably (13). Similarly, treating *N. sativa* increased β cell function markedly (14). Nehar demonstrated that *N. sativa* seeds extract can alleviate IR while also regulating blood glucose levels (15). Dalli demonstrated that different components of *N. sativa* seeds could inhibit α-amylase (*in vitro*) and intestinal glucose absorption (16).

**FIGURE 1:**
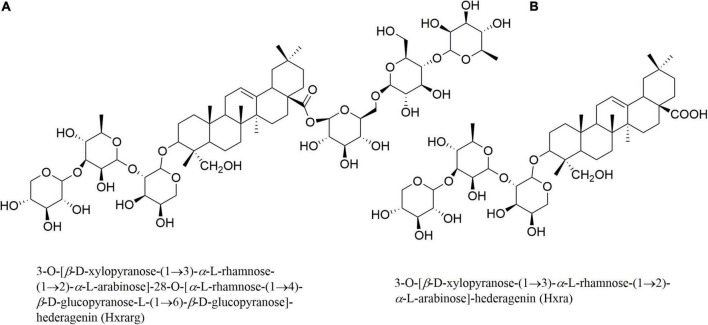
Structures of Hxrarg **(A)** and Hxra **(B)** from *Nigella sativa*.

The above results indicate that *N. sativa* seeds extract can ameliorate IR and T2DM. However, hypoglycemic effect and mechanism of its active compounds have yet to be reported. Multiple studies have shown that a variety of triterpenoids extracted from natural medicines have the effect of improving insulin resistance. Zhang et al. found that wacao pentacyclic triterpenoid saponins could increase insulin sensitivity, thereby improving insulin resistance (17). Hu et al. found that triterpenoid saponins isolated from *Stauntonia Chinensis* could improve insulin resistance by enhancing glucose uptake (18). Our research group systematically studied the chemical contents of *N. sativa* seeds in the early stages, and on this basis, screened the activity of 30 compounds. The results showed that two triterpenoids, 3-*O*-[β-D-xylopyranose-(1→3)-α-L-rhamnose-(1→2)-α-L-arabinose]-28-*O*-[α-L-rhamnose-(1→4)-β-D-glucopyranose-L-(1→6)-β-D-glucopyranose]-hederagenin (Hxrarg) and 3-*O*-[β-D-xylopyranose-(1→3)-α-L-rhamnose-(1→2)-α-L-arabinose]-hederagenin (Hxra) (Figure 1), could promote glucose uptake in 3T3-L1 adipocytes. Therefore, IR-3T3-L1 adipocytes were chosen to investigate the effects of Hxrarg and Hxra on glucose absorption and lipid metabolism, as well as the molecular mechanism of alleviating IR.

## Materials and methods

### Reagents and cells

3T3-L1 preadipocyte cell was got from Procell (Wuhan, China). Dulbecco’s Modified Eagle’s Medium (DMEM) and fetal bovine serum (FBS) were bought from Solarbio (Beijing, China). Oil red O staining solution was purchased from Beyotime Biotechnology (Shanghai, China). 3-isobutyl-1-methylxanthine (IBMX), dexamethasone (Dex), and insulin were obtained from Sigma-Aldrich (St. Louis, MO, United States). Sodium orthovanadate (Van) was purchased from Aladdin (Shanghai, China). Primer IRS, AKT, PI-3K, and GLUT4 were designed and synthesized by Thermo Fisher Scientific (Shanghai, China). *Evo M-MLV* RT Kit with gDNA Clean and TB Green TM Ex TaqTM II (Tli RNadeH Plus), Bulk kit were obtained from TaKaRa. Anti-IRS antibody, anti-phospho-IRS antibody, anti-AKT antibody, and anti-phospho-AKT antibody were bought from Cell Signaling Technology (Danvers, MA, United States). Anti-PI-3k antibody and anti-GLUT4 antibody were purchased from Abcam (Cambridge, United Kingdom).

### Cell culture

DEME high glucose medium containing 10% FBS, 100°U/mL penicillin and 100 g/mL streptomycin was added to the 3T3-L1 preadipocyte, then cultured at 37°C in an incubator with 5% CO_2_.

### MTT viability assay

The cell viability assay was consistent with the previously published approach (19). Briefly, 1.0 × 10^4^ cells were plated in 96-well-plates. Hxrarg and Hxra were added at increasing concentrations over the next 24 h. Each well received 20 μL of MTT reagent 4 h before the end of the experiments. Then 150 μL of dimethyl sulfoxide (DMSO) was added at the end to dissolve the formazan. At a wavelength of 570 nm, absorbance was recorded.

### 3T3-L1 preadipocyte differentiation

With minor changes, the 3T3-L1 preadipocyte differentiation was assessed by Yim’s method (20). 3T3-L1 preadipocyte were cultivated in DMEM and grown to confluence. After 48 h, the media was displaced to differentiation medium I, which contained 1°μM Dex, 10°μg/mL insulin and 0.5 mM IBMX. Then cultured for 48 h in DMEM with insulin (10°μg/mL) (differentiation medium II) before being transferred to fresh DMEM. Finally, the DMEM with 10% FBS was replaced every 2°days until mature adipocytes were differentiated.

### Oil red O stain

During differentiation, differentiation medium I, differentiation medium II, each medium exchange, Van (10°μM), varying concentrations of Hxrarg (100, 50, and 25°μM) and Hxra (25, 12.5, and 6.25°μM) were added to cells to exam effect of Hxrarg and Hxra on 3T3-L1 preadipocyte cells differentiation. Then medium was removed, cells were cleaned with PBS and fixed for 40 min with 10% paraformaldehyde. Then cells were washed with PBS, 50°μL of oil red O dye was added to each well for cell staining for 30°min. Then the cells were purged with pure water, photographed under fluorescence microscope to observe the lipid droplet generation in adipocytes. After that, the lipid was extracted with isopropanol to quantify by measuring the OD values at 492 nm.

### Intracellular triacylglycerol quantification

Triacylglycerol content was determined utilizing the procedure previously reported with slight adjustments (21). Van and different doses of Hxrarg or Hxra were added to the induction of differentiation of 3T3-L1 cells according to the aforesaid methods until they were differentiated into mature adipocytes. After washing, cells were collected and treated with ultrasound (300 W, repeat three times). In complete accordance with kit guidelines, intracellular TG content was determined.

### Establishment of IR-3T3-L1 cell model

3T3-L1 adipocytes cells that had been stimulated to differentiate and mature were used. Then, in model group, cells were treated with Dex (1°M), while the control group treated with DMEM. Then the residual glucose in control group and model group was detected every 12 h. The difference between the two groups was used to evaluate whether the IR cell model was successfully constructed and to determine the optimal treatment time of Dex. The two experimental groups were substituted with DMED at the same time once the IR model was successfully created. The glucose content in culture solution was measured every 12 h using the same procedure to establish the IR model’s duration.

### Measurement of glucose uptake

The control and model groups were changed with fresh DMEM, and other group was replaced with DMEM carrying Van (10°μM), Hxrarg (100, 50, and 25°μM), and Hxra (25, 12.5, and 6.25°μM) when the IR-3T3-L1 cell model was successfully established. The levels of glucose in the cell supernatant were detected by glucose oxidase-peroxidase according to instructions of glucose assay kit.

### Western blot

The cells were treated with above method (**Measurement of Glucose Uptake**) for 48 h later, Western Blot was carried out according to the reference (22).

### Quantitative real-time polymerase chain reaction

After IR-3T3-L1 cells have been treated with the above approaches, qRT-PCR was performed as previously mentioned (23). β-actin was regarded as normalization control. The sequences of primers were listed in Table 1.

**TABLE 1:** The sequences of primers.

Name	Primer	Sequence (5′→3′)
GLUT4	Forward	GTAACTTCATTGTCGGCATGG
	Reverse	AGCTGAGATCTGGTCAAACG
AKT	Forward	CACCGTGTGACCATGAACGAGTT
	Reverse	TGGCGACGATGACCTCCTTCTT
PI-3K	Forward	TCCTGGAATGTGCCCTCTTTCGT
	Reverse	TGACTGGCTGACTGCTGTGACT
IRS-1	Forward	GCCATCCCGAAAGCGAAGATCC
	Reverse	GCCTGTGCTCCTCCTGACTTGT
β-actin	Forward	AGCCTTGTAGGTACCCAACC
	Reverse	TCCACTCACCTGAGGTGCTGAA

### Statistical analysis

The data were analyzed by one-way ANOVA in SPSS 19.0 software. Results were shown as mean ± standard deviation. All column images were drawn with GraPhPad Prism 5.0.

## Results

### Effects of Hxrarg and Hxra on viability of 3T3-L1 preadipocytes

**FIGURE 2:**
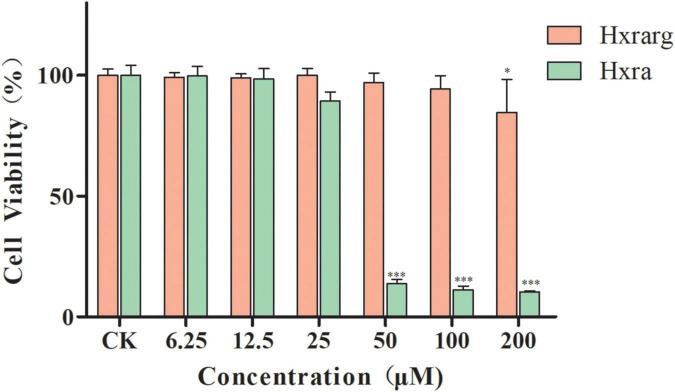
Viability effect of Hxrarg and Hxra on 3T3-L1 preadipocytes. Compared with control group, *P < 0.05, ***P < 0.001.

The survival rates of 3T3-L1 preadipocytes were greater than 95% when the concentration of Hxrarg was increased from 0 to 100°μM, as shown in Figure 2. The rate reduced dramatically when Hxrarg concentrations reached 200°μM. Hxra inhibited 3T3-L1 preadipocyte proliferation and reduced cell viability to less than 25% at a concentration of 50°μM, when concentration was less than 50°μM, survival rates were all above 90%.

### Differentiation and identification of 3T3-L1 preadipocytes

**FIGURE 3:**
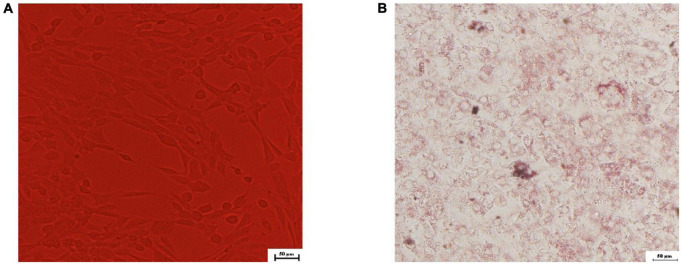
Microscopic view of 3T3-L1 preadipocytes differentiated to adipocytes. **(A)** 0th day; **(B)** oil red O staining in the 8th day.

Differentiation and identification of 3T3-L1 preadipocytes was analyzed qualitatively by oil red O in this research. There were no lipid droplets in cytoplasm of 3T3-L1 preadipocytes, which were irregularly polygonal or spindle-shaped (Figure 3A). A considerable number of lipid droplets were detected in cytoplasm after the differentiation medium I and II were added methodically and stained with oil red O. More than 80% of the cells had mature adipocyte characteristics (Figure 3B), showing that 3T3-L1 preadipocytes had been successfully differentiated.

### Effects of Hxrarg and Hxra on lipid accumulation in 3T3-L1 cells

**FIGURE 4:**
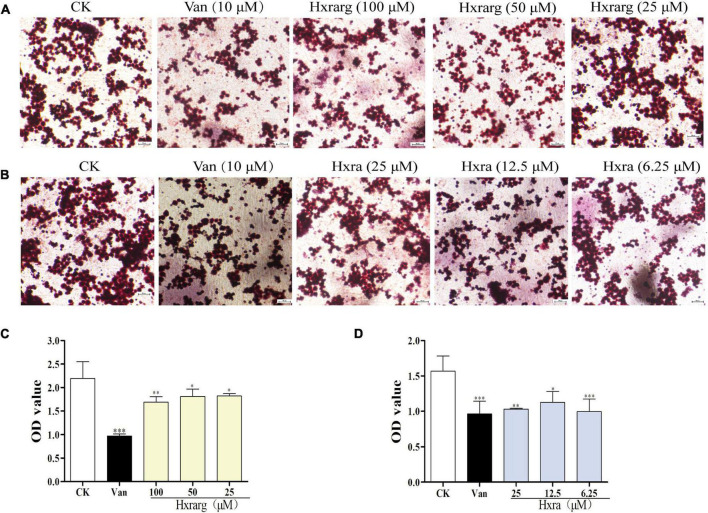
Inhibition of lipid droplet formation by Hxrarg **(A)** and Hxra **(B)**. Effects of Hxrarg **(C)** and Hxra **(D)** on lipid accumulation during 3T3-L1 differentiation. Compared with control group, *P < 0.05, **P < 0.01, ***P < 0.001.

In Figure 4, the cells in the control group have a substantial number of lipid droplets in cytoplasm, indicating that they are mature adipocytes. Lipid droplets were dramatically reduced in the positive group, demonstrating that cells treated with Van prevent lipid accumulation throughout the induction process. The creation of lipid droplets was greatly suppressed after cultured with Hxrarg or Hxra, which lower the amount of lipid droplets in the induction process and inhibit cell differentiation (Figures 4A,B). To validate the observation results, isopropanol was utilized to dissolve the lipid droplets in each treatment group, and the OD value was analyzed to determine the degree of differentiation. The less lipid droplets produced and the lower the level of adipocyte differentiation, the smaller the OD value. Compared with control group, lipid accumulation in 3T3-L1 cells in Van group and the Hxrarg and Hxra treatment groups at various concentrations decreased, as shown in Figures 4C,D. This clearly showed that the inhibitory effect on adipocyte differentiation. The findings were mostly in line with fluorescence microscopy’s trends.

### Effects of Hxrarg and Hxra on triacylglycerol content

**FIGURE 5:**
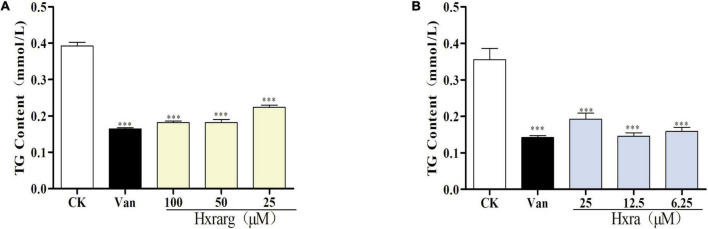
Effects of Hxrarg **(A)** and Hxra **(B)** on TG contents in 3T3-L1 cells differentiation. Compared with control group, ***P < 0.001.

The TG content in control group was higher, reaching 0.38 mM, as seen in Figure 5. The content in 3T3-L1 cells decreased markedly after Van treatment, to just 0.15 mM, indicating that Van could minimize TG accumulation during differentiation. In Hxrarg and Hxra treatment groups, the content was 0.16–0.21 mM, lower than that of control group, revealing that Hxrarg and Hxra could drastically lower the content of TG and block cell differentiation. This is also supported by the oil red O staining results mentioned above.

### Establishment of IR-3T3-L1 adipocyte model

**FIGURE 6:**
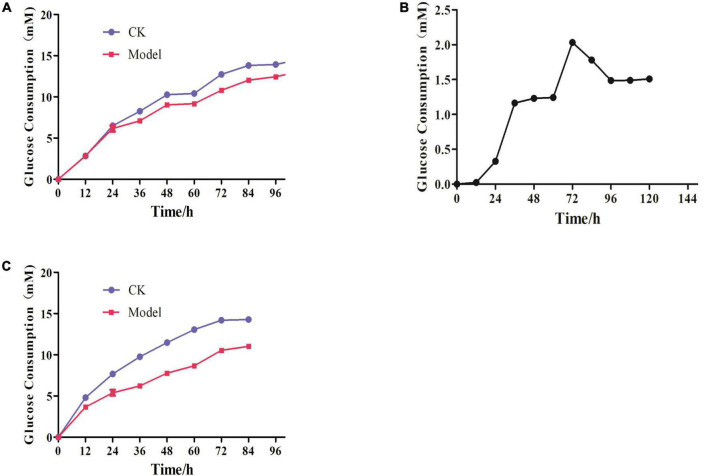
Establishment of IR-3T3-L1 adipocyte model. **(A)** Glucose consumption test during insulin resistance induced by Dex; **(B)** dex induced insulin resistance in adipocytes glucose consumption difference; **(C)** Stability of insulin resistance model.

In Figure 6A, there was no change in the amount of glucose residue between the control and the model groups after 24 h, and Dex had no effect on cells. The difference in glucose residual amount between the two groups gradually expanded after 48 h, implying that Dex was causing 3T3-L1 adipocytes to develop IR. The difference peaked at 72 h and then began to decline slowly. The optimal time of IR adipocyte model was 72 h after Dex treatment (Figure 6B). It was steady 60 h after the IR cell model was successfully constructed. Glucose content in supernatant of model group was decreasing after 72 h, compared with control group. As a result, 48 h was selected as administration time to study the effects of Hxrarg and Hxra on IR-3T3-L1 (Figure 6C).

### Effects of Hxrarg and Hxra on glucose uptake in IR-3T3-L1 cells

**FIGURE 7:**
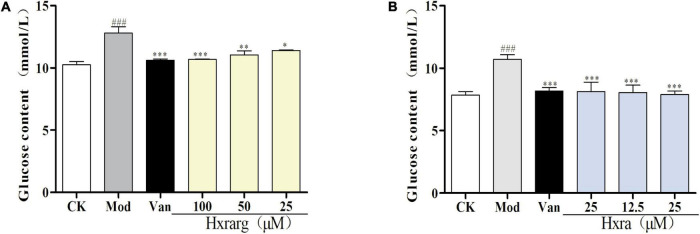
Glucose uptake effect of Hxrarg **(A)** and Hxra **(B)** on IR-3T3-L1 adipocytes. Compared with control group, ^###^*P* < 0.001; compared with model group, *P < 0.05, ***P < 0.001.

The glucose residual amount in model group was notably higher than that of control group, following Dex induction, as shown in Figure 7, displaying that the IR-3T3-L1 cell model was successfully established. The glucose content in the supernatant after Van treatment appreciably reduced compared with control group, reflecting that Van improved IR-3T3-L1 adipocyte glucose absorption. 3T3-L1 cells consume more glucose after being treated with Hxrarg and Hxra for 48 h, resulting in a considerable decline in glucose content, showing that Hxrarg and Hxra boost glucose uptake of IR-3T3-L1 adipocytes and ameliorate IR status.

### Expression of related protein in PI-3K/AKT signaling pathway

**FIGURE 8:**
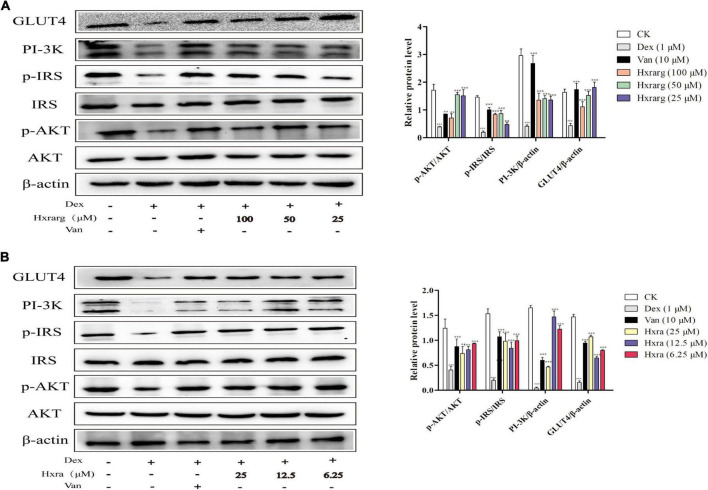
The effects of Hxrarg **(A)** and Hxra **(B)** on protein expression in IR adipocyte signaling pathway. Compared with the control, ^###^*P* < 0.001; compared with the model group, *P < 0.05, ***P < 0.001.

The IR cell model was successfully established after Dex treatment for 72 h, and PI-3K/AKT signaling pathway was impaired, preventing insulin signal transduction from continuing downstream or decreasing transmission efficiency. Phosphorylation of IRS and AKT, as well as expression levels of PI-3K and GLUT4 proteins, increased when the cells were stimulated with the Van. At different concentrations, Hxrarg and Hxra had no influence on IRS and AKT protein expression, but up-regulate expression of p-IRS, p-AKT, PI-3K, and GLUT4 (Figure 8). These findings reveal that Hxrarg and Hxra stimulates the PI-3K/AKT pathway to boost insulin signal transduction, ultimately improving IR.

### mRNA expression of insulin receptor substrate, AKT, PI-3K, and GLUT4

**FIGURE 9:**
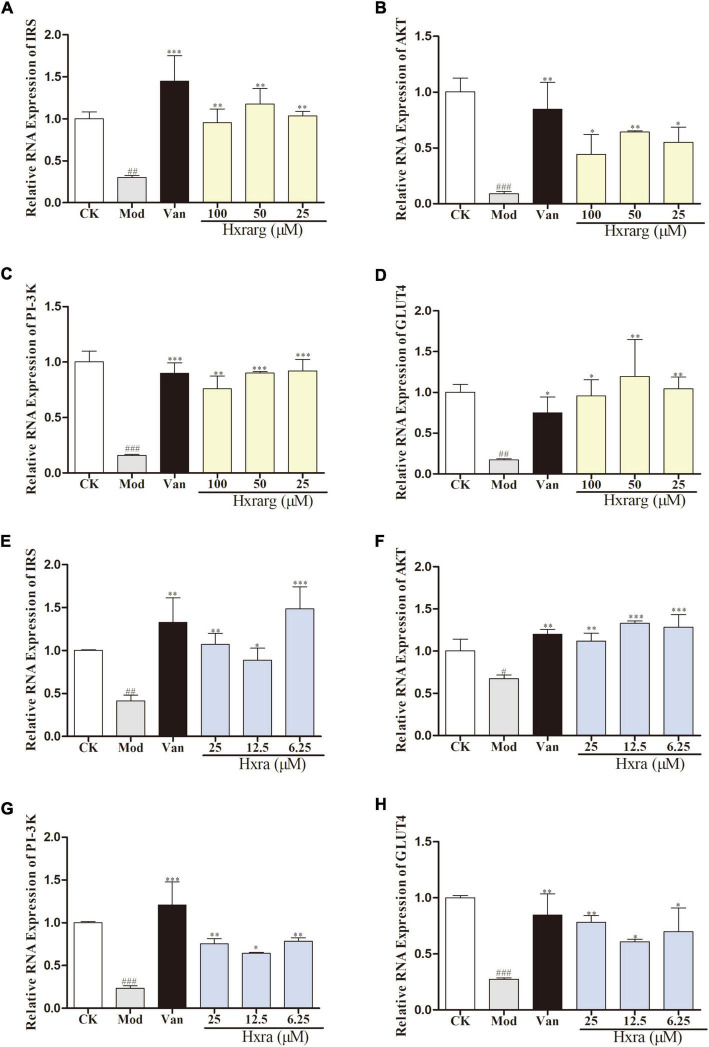
The effects of Hxrarg **(A–D)** and Hxra **(E–H)** on mRNA transcription. **(A,E)** IRS mRNA; **(B,F)** AKT mRNA; **(C,G)** PI-3K mRNA; and **(D,H)** GLUT4 mRNA. Compared with control group, ^##^*P* < 0.01, ^###^*P* < 0.001; compared with model group, *P < 0.05, **P < 0.01, ***P < 0.001.

The results of the mRNA relative expression were shown in Figure 9. Compared with control group, IRS, AKT, PI-3K, and GLUT4 mRNA expression in model group substantially decreased. Van could significantly enhanced expression of related genes. The expression of IRS, AKT, PI-3K, and GLUT4 mRNA were up-regulated in Hxrarg and Hxra group at different concentrations.

## Discussion

Effects on IR-3T3-L1 adipocytes of Hxrarg and Hxra isolated from *N. sativa* seeds were investigated in this paper. Hxrarg and Hxra could improve IR-3T3-L1 adipocytes, the mechanism may be by activating the related proteins on the insulin signal transduction pathway IRS/AKT/PI-3K in adipose tissue, thus promoting the glucose uptake of adipose cells.

Insulin resistance is one of the primary causes of T2DM, manifesting as impaired glucose intake, which is a common risk factor for many diseases, including lipid metabolism disorder, hypertension and coronary heart disease (24). Dex-induced IR model is easy to establish and has a strong correlation with clinical IR status *in vivo*. The mechanism of Dex-induced IR was investigated by many scholars. One theory is that IRS-1 expression is down-regulated, which is essential for GLUT4 translocation and plays a critical role in PI-3K inactivation (25). Another possibility is GLUT4 translocation damage, which is independent of insulin signaling (26). Liu (27) discovered that mature 3T3-L1 adipocytes treated for 48 h with 1°μM Dex could establish a stable IR-3T3-L1 model. Nie (28) found that inducing 3T3-L1 adipocytes with 1°μM Dex for 96 h reduced glucose absorption and maintained it for 36 h. In this study, 1°μM Dex could sharply reduce glucose consumption in supernatant after 72 h, and the IR model remained stable after 48 h.

Many studies have demonstrated that *N. sativa* seeds preparation could reduce blood glucose levels, increase insulin levels, improve insulin sensitivity, increase insulin immune reaction area, improve β cell number and diameter, reduce β cell damage and increase cell function, and so on (29, 30). However, there are few studies on the hypoglycemic molecular mechanism of *N. sativa* seeds. Most of the factors that cause IR interfere with the PI-3K activation of insulin signal. PI-3K/AKT signaling pathway mediates growth factor signals in organism growth and critical cell activities such as glucose homeostasis, lipid metabolism (31). Damage to the PI-3K/AKT pathway in numerous human tissues causes obesity, IR and T2DM. IR exacerbates the PI-3K/AKT signal transduction dysfunction, generating a vicious cycle. Insulin binds to the insulin receptor, causing IRS phosphorylation and the PI-3K/AKT signal cascade to be activated. By acting on downstream substrate molecules, phosphorylated AKT regulates cell growth and metabolism. AKT activation also promote the move of GLUT4 from storage vesicles to plasma membrane, increasing adipocyte glucose uptake. Expression of GLUT4 and glucose transport ability decrease when adipose tissue or cells produce IR (32, 33). Cheng (34) demonstrated that jatrorrhizine increase adipocyte glucose absorption, up-regulate protein expression of p-IRS, PI-3K, and p-AKT, increase the amount of GLUT4 on the cell membrane and improve IR. In IR-3T3-L1 adipocytes. In this paper, Hxrarg and Hxra enormously up-regulate gene expression in PI-3K/AKT signal pathway and protein expression levels of p-IRS, p-AKT, PI-3K, and GLUT4, thus improving adipocyte IR.

Adipose tissue is a key target organ for IR, intimately linked to disease. An increase in the number and volume of adipose cells leads to obesity. In addition, the larger the adipocytes, the less sensitive the tissues are to insulin. Treating glucose to overweight people can improve plasma insulin levels, reducing the physiologic impact of insulin. The target tissue’s insulin sensitivity returns when the patient loses weight (35). Therefore, adipocytes play an important role in energy storage and metabolism. Body weight gain and obesity can control by inhibiting preadipocyte differentiation or limiting the number of mature adipocytes. The effects of Van, Hxrag, and Hxra on adipocyte differentiation were examined in this study. Van dramatically inhibited the differentiation of 3T3-L1 preadipocytes. Korbecki found that Van can inhibit the differentiation of 3T3-L1 preadipocytes by reducing the activity of PTPase HA2 enzyme and increasing the amount of PTP1B (36). Hxrarg and Hxra also suppressed 3T3-L1 adipocyte adipogenesis and accumulation in this study.

More evidence supports that in clinical practice, a single TG index, TG/HDL-C ratio, or visceral fat index can be utilized to measure the IR of adult diabetes mellitus patients (37). 3T3-L1 cells have been widely used in researches of IR, glucose and lipid metabolism. Malgorzata (38) utilized 3T3-L1 cells to measure the effects of phospholipid cinnamate derivatives on IR and lipid metabolism. In this experiment, different concentrations of Hxrarg and Hxra could reduce TG content and increase glucose absorption in IR-3T3-L1 adipocytes. As a result, we suspect Hxrag and Hxrag can enable adipocytes better manage both glucose and lipid metabolism. Balbaa (39) reported that injecting *N. sativa* seeds oil into T2DM rats significantly increased insulin receptor gene expression while decreasing blood giucose, liposome composition, and serum insulin/insulin receptor ratio. Furthermore, our experimental results have been verified by our previous studies. In T2DM mice, *N. sativa* seeds polysaccharide (NSSP) reduces blood glucose and improves aberrant blood lipid levels (40).

**FIGURE 10:**
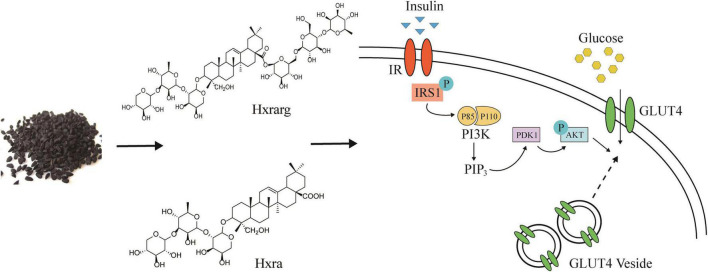
Hxrarg and Hxra activate PI-3K/AKT signaling pathways to promote the translocation of GLUT4.

Nearly 100 phytochemicals have been discovered in *N. sativa* seeds, but many have yet to be biologically verified (41). Triterpenoids have strong antioxidant properties, block the creation of advanced glycation end products, participate in various molecular mechanisms of diabetes and its complications, and inhibit or express a number of genes involved in the evolution of diabetes and its complications (42, 43). Rb2, the triterpenoid saponin found in the largest abundance in ginseng, increased glucose absorption in 3T3-L1 cells *in vitro*, mostly through the IRS/PI-3K/AKT pathway, and was unaffected by insulin receptor -β subunit (IRβ) (44). Our experimental results also confirmed that, as triterpenoids, Hxrarg, and Hxra reverse the damaged state of IRS/PI-3K/AKT signaling pathway and promote the absorption of glucose by 3T3-L1 cells. The mechanism diagram is shown in Figure 10. Their activity in promoting glucose absorption is similar, but the effective dose of Hxrarg is four times that of Hxra, this may be determined by the structure of the compound. Hxrarg and Hxra had the same parent nucleus, and their structural distinction is mainly caused by the three glycosyl on the carboxyl group of Hxrarg, we speculated that the carboxyl group may be the active group of Hxrarg and Hxra promoting glucose absorption in adipocytes, with reduced activity when the carboxyl group is connected to the glycosyl. Furthermore, it has been discovered that triterpenoids can lower blood glucose in a specific way, or they can work jointly in numerous ways and across multiple targets (45), which gives us new ideas for studying the hypoglycemic mechanism of Hxrarg and Hxra. Therefore, it is necessary to investigate the effects of Hxrarg and Hxra on other insulin-sensitive tissues, including skeletal muscle and liver, and the glucose effects in animal models of T2DM in the future.

## Conclusion

Effects on IR-3T3-L1 adipocytes of Hxrarg and Hxra isolated from *N. sativa* seeds were investigated in this paper. Hxrarg and Hxra could ameliorate IR-3T3-L1 adipocytes and promote glucose uptake of IR-3T3-L1 adipocytes, the mechanism may be by promoting the expression of IRS, AKT, PI-3K, and GLUT4 mRNA, promoting the expression of PI-3K, p-AKT, p-IRS-1, and GLUT4 proteins, thus activating IRS/AKT/PI-3K signaling pathway in adipose tissue. In addition, Hxrarg and Hxra inhibited differentiation of 3T3-L1 preadipocytes and reduced accumulation of TG. These findings maybe facilitate the development of functional foods based on *N. sativa* seeds for diabetic therapy.

## Data availability statement

The datasets presented in this study can be found in online repositories. The names of the repository/repositories and accession number(s) can be found in the article/supplementary material.

## Author contributions

JW: conceptualization, investigation, and writing – original draft. BW: writing – original draft and data curation. YC: investigation and data curation. QW and AA: formal analysis. LC: writing – review and editing. XX: editing. WK: project administration and editing. All authors contributed to the article and approved the submitted version.
